# Supplementary material for: Climate as a Predictive Factor for Invasion: Unravelling the Range Dynamics of Carpomya vesuviana Costa
Source: Insects. 2024 May 21;15(6):374. doi: 10.3390/insects15060374 (PMC11203509; doi:10.3390/insects15060374)
Supplement: Supplementary file 1 [file insects-15-00374-s001.zip › insects-3002720-supplementary.pdf]

Table S1 Distribution points for *Carpomya vesuviana*

Number	Longitude	latitude
1	73.61	22.77
2	75.46	29.1
3	76.58	28.08
4	76.62	28.18
5	76.15	28.28
6	77.03	28.47
7	76.13	28.78
8	75.7	16.83
9	77.21	23.17
10	79.09	21.16
11	74.65	19.38
12	74.29	30.12
13	71.68	29.4
14	74.31	32.17
15	73.02	26.28
16	75.38	26.97
17	80.25	13.06
18	78.1	9.52
19	72.82	24.12
20	77.21	28.67
21	77.21	28.57
22	101.16	12.41
23	102.62	12.36
24	99.95	13.1
25	101.49	6.25
26	100.08	7.62
27	101.27	6.89
28	98.99	18.79
29	101.17	6.32
30	70.55	31.49
31	71.68	29.4
32	51.22	29.11
33	52.85	32.75
34	89.1	42.6
35	88.6	42.8
36	90.3	43.8
37	57.29	-20.13
38	14.17	41.89
39	17.27	44.29
40	44.2	14.567
41	37.1	3.1
42	23.79	55.5

43	32.9	54.2
44	50.844	30.25
45	51.796	31.757
46	71.386	32.947
47	71.386	29.06
48	75.749	33.185
49	75.749	31.519
50	75.987	29.853
51	71.228	22.874
52	72.973	26.046
53	74.797	27.95
54	75.669	30.171
55	77.176	31.122
56	80.032	27.395
57	81.301	27.275
58	101.05	13.752
59	98.909	9.152
60	100.574	7.09
61	58.855	26.601
62	47.75	33.581
63	48.782	33.978
64	81.459	26.522
65	76.701	22.477
66	76.304	20.811
67	77.573	18.194
68	78.604	17.084
69	76.652	16.29
70	78.604	17.084
71	76.652	16.29
72	78.604	17.084
73	76.652	16.29
74	78.604	13.594
75	77.256	12.245
76	79.159	11.135
77	77.811	9.311
78	77.335	8.755
79	90.026	22.715
80	98.909	19.622
81	99.543	19.225
82	99.781	14.228
83	101.844	13.356
84	99.861	8.359
85	101.526	6.376
86	49.575	33.185

87	50.13	32.55
88	53.62	30.171
89	89.263446	42.675243
90	89.153509	42.954974
91	88.611261	42.847436
92	88.612451	42.849311
93	89.76699	42.754458
94	89.609882	42.773104
